# A new species of the *Dendropsophus decipiens* Group (Anura: Hylidae) from Northeastern Brazil

**DOI:** 10.1371/journal.pone.0248112

**Published:** 2021-07-14

**Authors:** Rogério Ferreira de Oliveira, Felipe de Medeiros Magalhães, Bernardo Franco da Veiga Teixeira, Geraldo Jorge Barbosa de Moura, Clara Ribeiro Porto, Francisco Péricles Branco Bahiense Guimarães, Ariovaldo Antônio Giaretta, Moacir Santos Tinôco

**Affiliations:** 1 Prograrma de Pós-graduação em Ecologia da Universidade Federal Rural de Pernambuco (UFRPE), Recife, Pernambuco, Brazil; 2 Programa de Pós-Graduação em Ciências Biológicas, Centro de Ciências Exatas e da Natureza, Universidade Federal da Paraíba (UFPB), João Pessoa, Paraíba, Brazil; 3 Laboratório de Taxonomia e Sistemática de Anuros Neotropicais, Instituto de Ciências Exatas e Naturais do Pontal, Universidade Federal de Uberlândia (UFU), Ituiutaba, Minas Gerais, Brazil; 4 Laboratório de Estudos Herpetológicos e Paleoherpetológicos da Universidade Federal Rural de Pernambuco (LEHP-UFRPE), Recife, Pernambuco, Brazil; 5 Centro de Ecologia e Conservação Animal (ECOA), Programa de Pós-Graduação em Território, Ambiente e Sociedade—Universidade Católica do Salvador (UCSAL), Salvador, Bahia, Brazil; Nanjing Agricultural University, CHINA

## Abstract

We describe a new species of the *Dendropsophus decipiens* Group, morphologically most resembling *D*. *haddadi* but genetically more closely related to *D*. *oliveirai* and likely endemic from the Atlantic Forest biome, northeastern Brazil. The new species can be distinguished from all species of the *D*. *decipiens* Group based on the combination of morphological features, advertisement call and phylogenetic position based on mitochondrial DNA gene sequences. The new species emits simple calls in series of 3–9 notes, each with 9–29 pulses, and dominant frequency varying from 5578–6422 Hz, and exhibit a minimum of 8% genetic distance (*16S* mitochondrial gene) in comparison to its congeners. The new taxa represent the sixth species of the *D*. *decipiens* Group, which likely harbors more undescribed taxa, corroborating the view that Neotropical species richness is fairly underestimated.

## Introduction

The genus *Dendropsophus* Fitzinger, 1843 currently comprise 109 species broadly distributed across Neotropical rainforests and open areas from southern Mexico to northern Argentina and Uruguay, east of Andes [1]. Based on the recently published total evidence analysis of [2], combining phenomic and molecular datasets, nine species groups are currently recognized within this genus: *D*. *ruschii*, *D*. *decipiens*, *D*. *parviceps*, *D*. *molitor*, *D*. *columbianus*, *D*. *marmoratus*, *D*. *minutus*, *D*. *leucophyllatus*, and *D*. *microcephalus*. These authors made many substantial changes to the systematics of this genus with respect to that of [3], including the first molecular assessment for species in the *D*. *decipiens* Group. Additionally, they also uncovered the existence of eight divergent genetic lineages indicated as *D*. *decipiens* (I–VIII), which are paraphyletic relative to samples of *D*. *haddadi* and *D*. *oliveirai*. Moreover, they placed *D*. *bromeliaceus* within the *D*. *decipiens* Group, which was originally not assigned to any of the previously recognized Groups at that time [3,4].

The *D*. *decipiens* Group currently comprises five species (*sensu* [2]): *D*. *berthalutzae* (Bokermann, 1962), *D*. *bromeliaceus* Ferreira et al., 2015, *D*. *decipiens* (Lutz, 1925), *D*. *haddadi* (Bastos and Pombal, 1996) and *D*. *oliveirai* (Bokermann, 1963), which exhibits as synapomorphies 11 phenomic characters, small SVL (combined SVLs range from 13.0–21.4 mm for males and 18.0–24.0 mm for females) and a brownish or pale yellow dorsum coloration with a frame-like pattern [2]. Species of the *D*. *decipiens* Group are distributed along eastern Brazil and mostly associated with the Atlantic Forest [1], except for *D*. *oliveirai* which is also found in ecotonal areas of Caatinga (a seasonally dry tropical forest) and Atlantic Forest in northeastern Brazil from Bahia to Rio Grande do Norte States [1,5,6]. More specifically, *D*. *berthalutzae* occurs along a narrow Atlantic Forest zone in southeastern Brazil from Paraná to Espírito Santo States, terminals assigned to *D*. *decipiens* occur across Bahia, Ceará, São Paulo, Minas Gerais, Rio de Janeiro and Espírito Santo States, *D*. *haddadi* occurs from the Espírito Santo to Pernambuco States, and *D*. *bromeliaceus* is currently only known from rocky outcrops in the municipality of Santa Teresa, Espírito Santo, southeastern Brazil [1,2,4].

During field work in Atlantic Forest remnants of Pernambuco State, we collected individuals of the *Dendropsophus decipiens* Group that could not be assigned to any of the five currently recognized species, and genetically matches the clade *D*. *decipiens* V, highlighted as a candidate new species by [2]. The high levels of morphological similarity and intraspecific variation hinders the advance of taxonomic resolutions among *Dendropsophus* species [2,7,8], making the use of multiple lines of evidence highly desirable to elucidate patterns of cryptic diversity within the genus. In this sense, we combine morphological, acoustic and molecular data to describe the clade *D*. *decipiens* V of [2] as a new species, representing the sixth species of *D*. *decipiens* Group likely associated to the Atlantic Forest, and the first with the type locality within the Pernambuco Endemism Center, which represents one of the most endangered Atlantic Forest remnants in Brazil [9,10].

## Material and methods

### Study area and reference material

**Fig 1:**
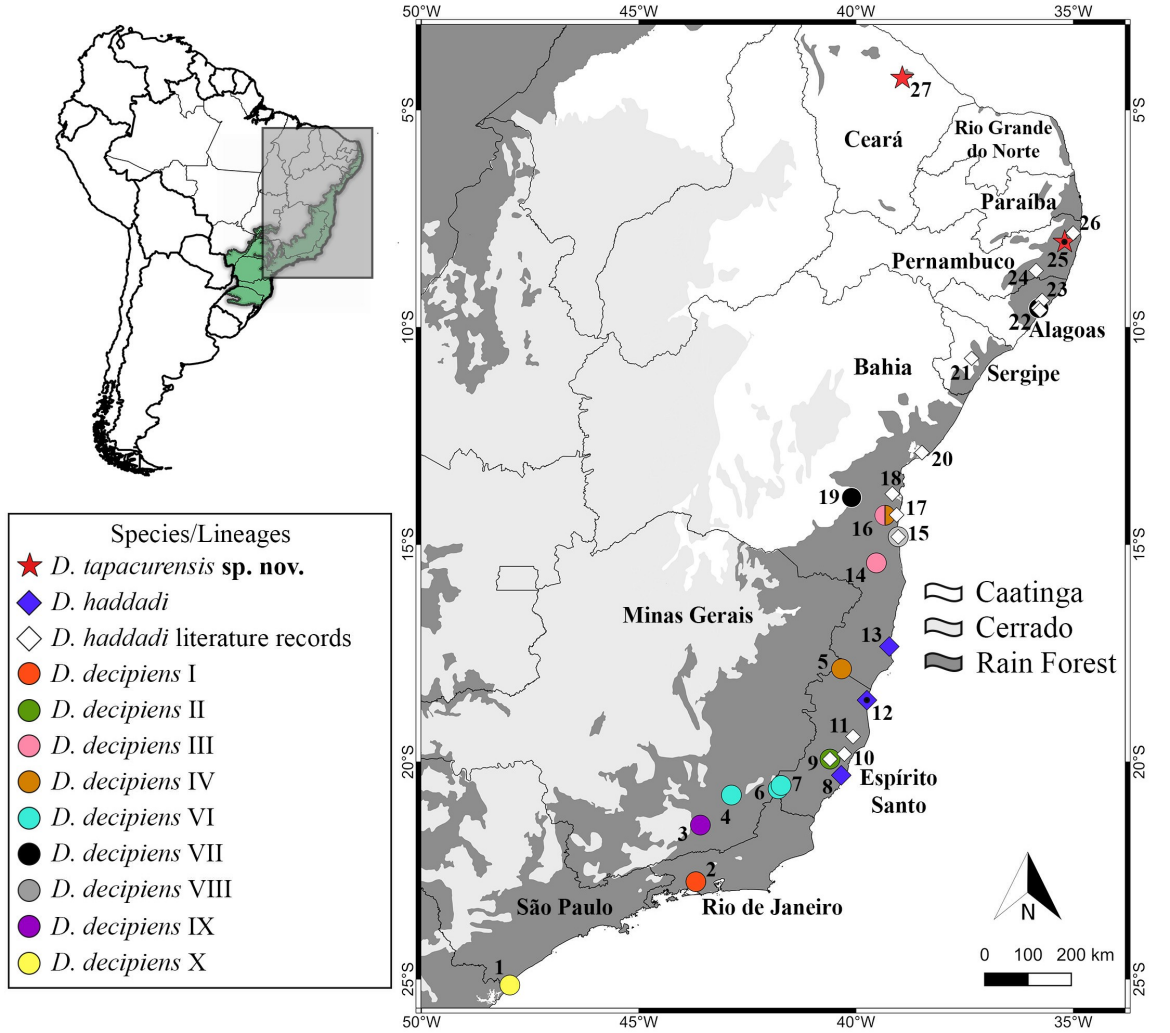
Geographic distribution of the new species. We also provide the geographic distribution of all clades labelled as *Dendropsophus decipiens* [2] and literature-based records of *D*. *haddadi* in the states of Alagoas [14,15], Bahia [16–20], Espírito Santo [17,21], Pernambuco [22,23] and Sergipe [24]. Municipalities: São Paulo State: (1) Cananéia; Rio de Janeiro State: (2) Seropédica; Minas Gerais State: (3) Santos Dumont, (4) Juiz de Fora, (5) Nanuque; Espírito Santo State: (6) Dores do Rio Preto, (7) Ibitirama, (8) Vitória, (9) Santa Teresa, (10) Aracruz, (11) Linhares, (12) Conceição da Barra (*D*. *haddadi* type locality); Bahia State: (13) Prado, (14) Camacan, (15) Ilhéus, (16) Aurelino Leal, (17) Itacaré, (18) Igrapiúna, (19) Jequié, (20) Mata de São João; Sergipe State: (21) Areia Branca; Alagoas State: (22) Maceió, (23) Maceió (Catolé and Ferrão Velho); Pernambuco State: (24) Lagoa dos Gatos, (25) São Lourenço da Mata (*D*. *tapacurensis* type locality), (26) Igarassu; Ceará State: (27) Guaramiranga.

We conducted field work on July 2018 at two conservation units: the Buchada Forest (100 ha) (8°2’26.13"S, 35°12’0.43"W; 122 m above sea level [a.s.l.], DATUM WGS84) and the Camocim Forest (200 ha) (8°1’59.75"S, 35°12’3.79"W; 131 m a.s.l., DATUM WGS84). These two conservation units compose the Tapacurá Ecological Station, a protection unit administered by the Universidade Federal Rural de Pernambuco, located at São Lourenço da Mata municipality, Pernambuco State, Brazil (Fig 1). The area is characterized by semi deciduous seasonal Atlantic Forest fragments [11], the climate is monsoon type (Am) according to classification by [12], and the rainy season occurs from February to September with average annual precipitation of 1.900 mm [13].

We collected a total of 21 adult specimens in the studied area (permit IBAMA/RAN 087/07). Specimens were euthanized with 5% lidocaine, fixed in 10% formaldehyde, preserved in 70% ethanol and deposited at Coleção Herpetológica e Paleoherpetológica of the Universidade Federal Rural de Pernambuco, Recife, Brazil (CHP-UFRPE 5697–5717). Locality data is given in Fig 1. For comparisons, we evaluated specimens of *D*. *berthalutzae* from Paranapiacaba, São Paulo State (topotypes), *D*. *decipiens* from Duas Barras (distant 125 km from type locality), Rio de Janeiro State, *D*. *haddadi* from Santa Teresa (paratopotypes) and Sooretama (distant 65 km from type locality), Espírito Santo State, and *D*. *oliveirai* from Maracás, Bahia State (topotypes), all of which are housed at Collection of frogs (AAG-UFU) at Universidade Federal de Uberlândia, Uberlândia municipality, Minas Gerais State (S1 Appendix). Other specimens examined by us are listed in S1 Appendix. Institutional abbreviations followed [25]. Tissue samples were made available by the tissue sample collection of the Laboratório de Anfíbios e Répteis da Universidade Federal do Rio Grande do Norte (CLAR–UFRN, AAGARDA) Amphibian collection of Universidade Federal de Juiz de Fora (CAUFJF), and Herpetological collection of Museu de História Natural, Universidade Federal de Alagoas (MUFAL).

### Morphometry

We measured specimens using a Digimess digital caliper (to the nearest 0.1mm). Eight measurements followed [26] terminology: snout-vent length (SVL), head length (HL), head width (HW), eye diameter (ED), tympanum diameter (TD), eye-nostril distance (END), foot length (FL), and shank length (SL). For the other two characters, we followed [27]: hand length (HAL) and thigh length (THL). Webbing formula followed [28].

### Acoustics

We recorded advertisement calls with a TASCAM DR40 digital recorder set at 44.1 kHz and resolution of 16 bits, coupled to a Yoga HT81 directional microphone. Measurements were analyzed using Raven Pro v1.5 for Windows from The Cornell Lab of Ornitology [29]; spectrogram settings were Hann, window size = 1024 samples, 3 dB bandwidth = 270 Hz, Overlap = 85%, hop size = 0.792, DFT size = 1024 samples, and grid spacing = 46.9 Hz. All other settings followed the ‘default’ of Raven. Sound figures were obtained in the Seewave package v1.5.9 [30], on the R platform v3.6.1 [31]; Seewave settings were Hanning window, 256 points resolution (FFT), and 85% of overlap. Call terminology follows [32], using a note-centered approach. The recording files were deposited at the Sonoteca Coaxar of the Coleção Herpetológica e Paleoherpetológica of the Universidade Federal Rural de Pernambuco, Recife, Brazil (SCLEHP 18–28; see S2 Appendix).

### Molecular analysis

We assembled a total of 18 tissue samples from representatives of the *Dendropsophus decipiens* Group (e.g., *D*. *berthalutzae*, *D*. *decipiens*, *D*. *haddadi* and *D*. *oliveirai*), including five paratopotypes of the new species. We extracted genomic DNA from liver tissues and amplified the mitochondrial H-strand transcription unit 1 (*H1*; which include segments of the *12S* and *16S* ribosomal genes, and the intervening valine-*tRNA*) using primers and Polymerase Chain Reactions (PCRs) protocols provided by [3]. Total DNA was extracted from tissue samples using Kasvi’s Mini Spin DNA Extraction Kit following the protocols described in the kit manual, except for the addition of QIAGEN’s Tissue Lysis Buffer in the first DNA extraction step. PCR products were then purified using Invitrogen’s PureLink ™ Genomic DNA Mini Kit following the protocol described in the kit without any modification. Purified PCR products were sequenced using the BigDye Terminator v.3.1 Cycle Sequencing Kit. We assembled a complete *H1* segment (~2400 base pairs [bp]) for two samples, while the remaining 19 samples had at least the final *16S* segment (ca. 550pb; primers 16Sar–br) from [33] sequenced. We checked sequencing quality and edited chromatograms in the program Geneious v1.8.7 [34].

To infer the phylogenetic relationships of the new species, we created a final alignment dataset for 233 terminals including our 18 sequenced individuals plus 193 GenBank sequences of *Dendropsophus* species that had the complete *H1* segment available or are members of the *D*. *decipiens* Group, encompassing individuals from all species groups or phenetic clades proposed for the genus [2,3,35,36]. As outgroups, we selected 22 terminals including species of genus *Lysapsus*, *Phyllodytes*, *Pseudis*, *Scarthyla*, *Scinax*, *Sphaenorhynchus*, and *Xenohyla*. We aligned sequences using the E-INS-I strategy of MAFFT algorithm [37] also implemented in Geneious v1.8.7 [32], and used the resulting alignment with 2608 bp (gaps included) as input for phylogenetic analyses. We generated hypotheses of phylogenetic relationships among species of *Dendropsophus* using maximum likelihood in RAxML v8.2.12 [38] and Bayesian inference in MrBayes v3.2.7 [39], implementing the GTR+I+G substitution model as suggested by the Akaike Information Criterion [40] in jModeltest version 2.1.6 [41]. We obtained maximum likelihood tree estimates with nodal support assessed via 1000 rapid bootstrap replicates. For Bayesian inference, we ran MrBayes analysis for 20x10^6^ generations, with two parallel runs and eight chains each, sampling every 2000 steps. We assessed runs convergence by examining the average standard deviation of the split frequency between runs (< 0.01) and effective sample size (> 200) with Tracer v1.7 after discarding the initial 20% generations as burn-in, and drew phylogenetic trees using FigTree v1.4.2 [42]. We ran both analyses using the resources provided by CIPRES Science Gateway platform [43]. Finally, we computed between-group mean distances between the new species and species/lineages in the *D*. *decipiens* Group using Tamura & Nei [44] corrected p-distances with MEGA v7.0 [45]. Prior to this analysis, we trimmed our alignment to fit the shortest sequence available, resulting in a 400 bp alignment (comprising the final *16S* segment) employed to calculate distances. GenBank accession numbers for all sequences used by us are given in S3 Appendix.

#### Nomenclatural acts

The electronic edition of this article conforms to the requirements of the amended International Code of Zoological Nomenclature, and hence the new names contained herein are available under that Code from the electronic edition of this article. This published work and the nomenclatural acts contained have been registered in ZooBank, the online registration system for the ICZN. The ZooBank LSIDs (Life Science Identifiers) can be resolved and the associated information viewed through any standard web browser by appending the LSID to the prefix "http://zoobank.org/". The LSID for this publication is: urn:lsid:zoobank.org:pub:4117F1FC-D5BD-439F-B210-6127A5AA07D9. The electronic edition of this work was published in a journal with an ISSN has been archived and is available from the following digital repositories: PubMed Central and LOCKSS.

## Results

**Fig 2:**
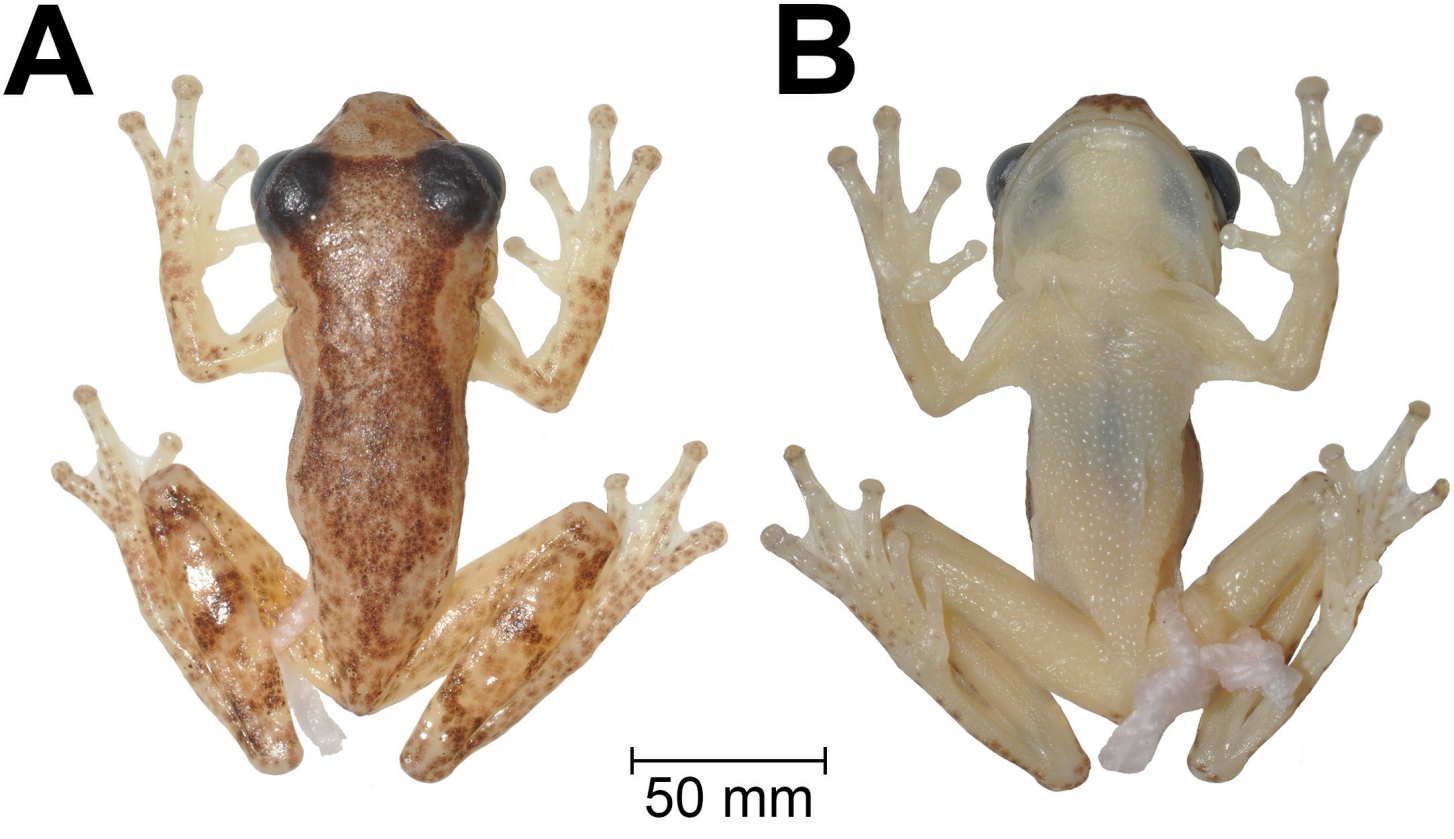
Holotype of *Dendropsophus tapacurensis* sp. nov. (CHP-UFRPE 5709). (A) dorsal and (B) ventral views of body.

**Fig 3:**
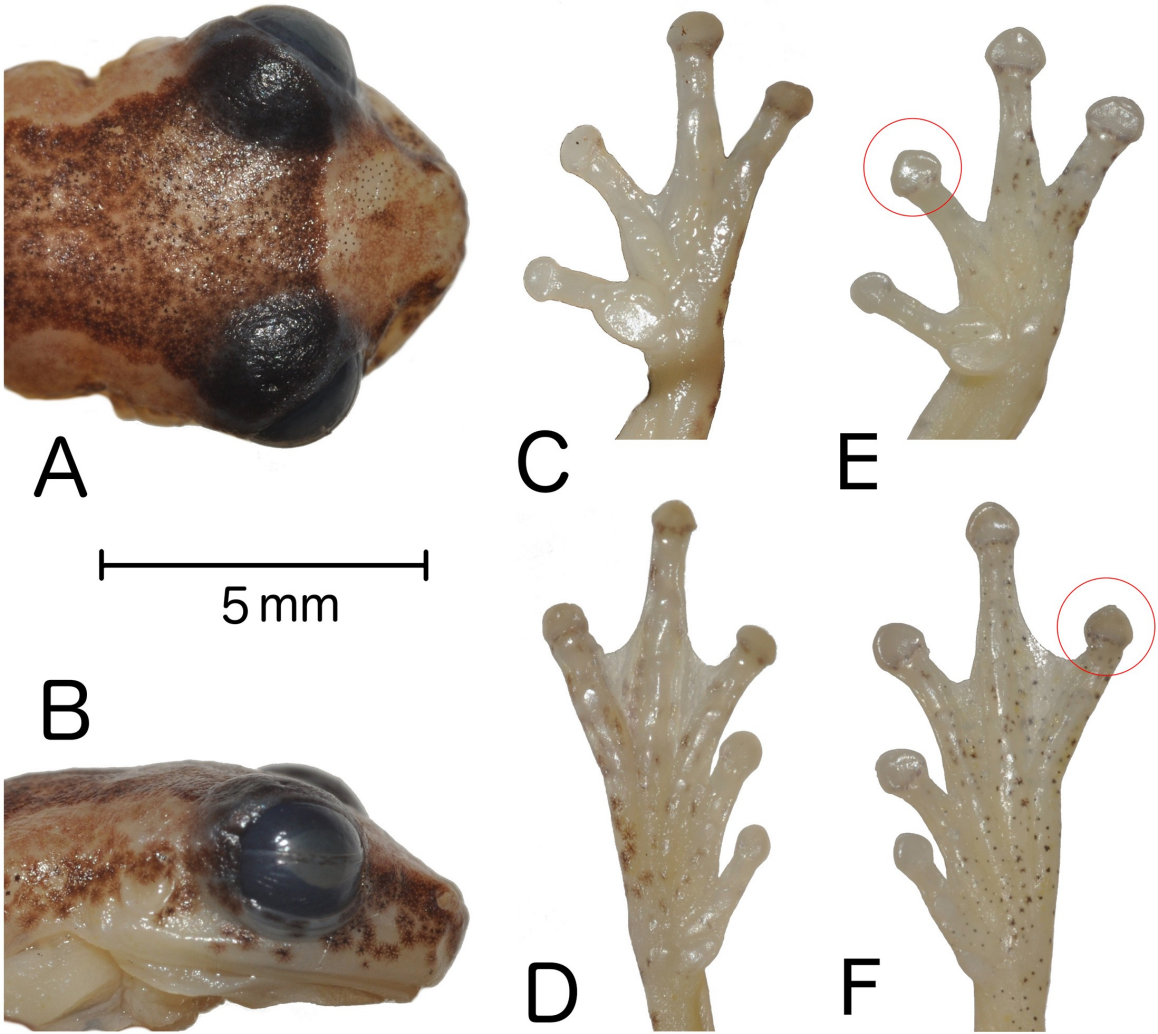
Holotype of *Dendropsophus tapacurensis* sp. nov. (CHP-UFRPE 5709). (A) Dorsal and (B) profile views of head. Views of (C) hand and (D) foot. View of female CHP-UFRPE 5703 (E) hand and (F) foot, evidencing the presence of pointed discs.

**Fig 4:**
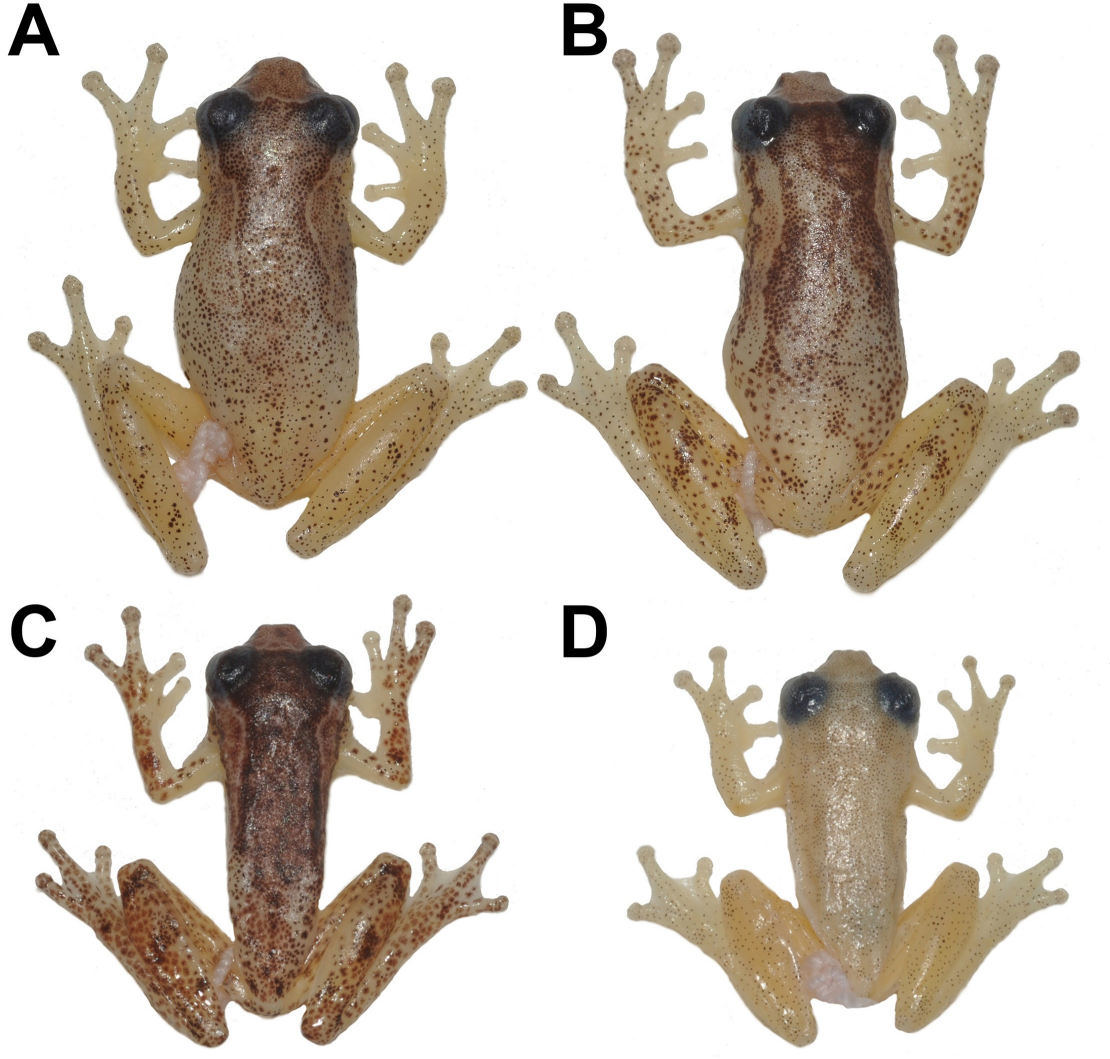
Specimens of *Dendropsophus tapacurensis* sp. nov. depicting differences in pigmentation patterns and size. Females (A) CHP-UFRPE 5712 and (B) CHP-UFRPE 5703; and males (C) CHP-UFRPE 5702 and (D) CHP-UFRPE 5713.

**Fig 5:**
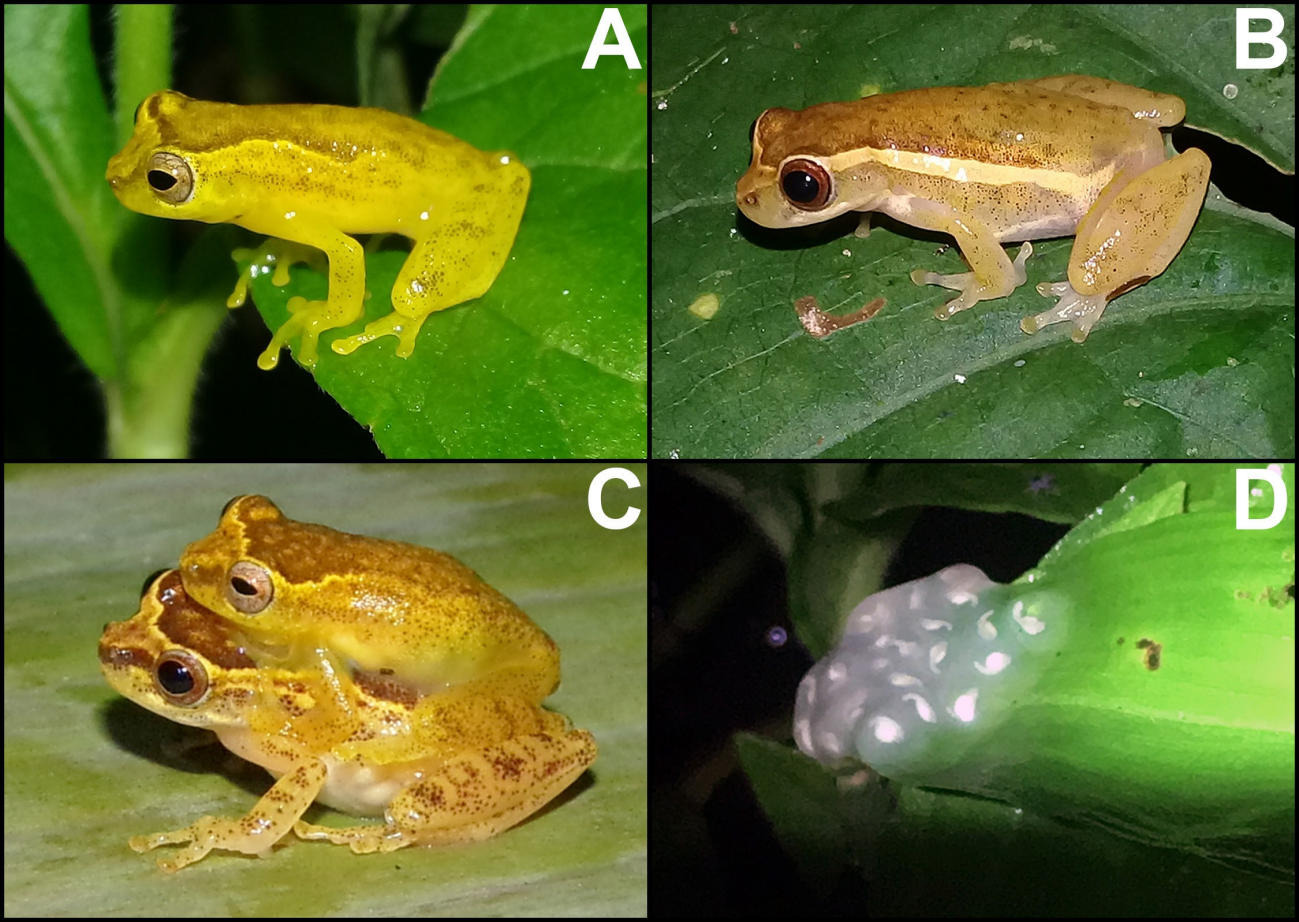
Live specimens of *Dendropsophus tapacurensis* sp. nov. from type locality. (A) adult male; (B) adult female; (C) amplectant pair; (D) egg mass deposited on leaves overhanging at standing water.

*Dendropsophus tapacurensis* sp. nov. (Figs 2–5) urn:lsid:zoobank.org:act:A3171D2B-5A34-4EE1-ABE9-732FD8F01BC9.

### Holotype

Adult Male (CHP-UFRPE5709), collected on 13 July 2018 at the Estação Ecológica do Tapacurá, Municipality of São Lourenço da Mata, Pernambuco State, Brazil (8°2’26.13"S, 35°12’0.43"W; 122 m a.s.l., DATUM WGS84) by Rogério F. de Oliveira and Felipe de M. Magalhães.

### Paratopotypes

Eighteen adult males CHP-UFRPE 5697–5702, CHP-UFRPE 5704–08, CHP-UFRPE 5710–11, CHP-UFRPE 5713–17 and two egg-bearing females CHP-UFRPE 5703 and CHP-UFRPE 5712, collected along with the holotype.

### Referred material

Two genetic vouchers (CFBHT 11304, 11322) from Guaramiranga municipality, Ceará State (Fig 1), originally assigned as *Dendropsophus decipiens* “V” by [2].

### Diagnosis

We assigned the new species to the genus *Dendropsophus* and specifically to the *D*. *decipiens* Group based on our phylogenetic analysis results (see below). In addition, the new species morphologically resembles other species of the *D*. *decipiens* Group (especially *D*. *haddadi*) exhibiting a frame-like coloration pattern (with interorbital and dorsolateral bands) and lay eggs on leaves overhanging temporary ponds (Fig 5D), features common among species of this Group [3].

*Dendropsophus tapacurensis* sp. nov. can be distinguished from congeners by the combination of the following features: (1) small size, adult males 15.5–17.6 mm SVL (mean 16.4 mm) and adult females 19.5–20.7 mm SVL (mean 20.1 mm); (2) head wider than long; (3) vocal sac single, subgular, and light yellow (in life); (4) the presence of transversal bars on shanks; (5) dorsolateral region delimited by an irregular light brown strip that extends from the posterior region of the eye towards the inguinal area; (6) presence of a triangular-shaped mark in the loreal region with the same coloration of the dorsolateral strip; (7) advertisement call with only one type of note, emitted in sequences of three to nine notes; (8) notes with 9 to 29 pulses; (9) pulse repetition rate ranging from 143 to 368 pulses/s; and (10) dominant frequency ranging from 5578–6422 Hz.

### Holotype description

Adult male. Proportions of body parts in relation to SVL (16.5 mm): head length 0.31; head width 0.36, eye diameter 0.17, tympanum diameter 0.06, hand 0.32, foot 0.4, femur 0.45, tibia 0.51. Head wider than long; snout truncated, discretely mucronate in dorsal view and rounded in lateral view; snout tip (mucronate condition) perceivable in lateral view at the level of nostrils; canthus rostralis rounded, slightly curved; loreal region slightly concave; nostrils slightly protuberant, directed dorsolaterally; interorbital area flat; eyes large and protuberant; pupil horizontally elliptical; lower eyelid mostly transparent, its free border pigmented as the upper eyelid; supratympanic fold barely visible, not extending beyond tympanum; tympanum distinct, circular, annulus barely defined dorsally; choanae oval; vomerine teeth small, only perceptible when probed; tongue cordiform, notched behind, posterior ¼ free from mouth floor; vocal slits well defined; vocal sac developed, single, subgular, extending over chest; forearm slender than arm; axillary membrane reaching half arm; out margin of forearm smooth; fingers bearing circular discs, albeit disc of finger IV slightly pointed; relative length of fingers I < II <IV < III; second subarticular tubercles well-defined, shallow, round on fingers I, II III and cordiform on IV; inner metacarpal tubercle flat, shallow, fused to the adjacent subarticular; outer metacarpal tubercle indistinct; nuptial pads covering the prepolical area, perceived as discrete asperities; webbing formula ItraceII2–3III21/2–2+IV; hind limb long and slender; no tarsal fold; no calcar ornament; toe discs I, II, III and V circular, IV discretely pointed; relative lengths of toes I < II < IV < III < V; subarticular tubercles round and shallow; inner metatarsal tubercle shallow, flat, elliptical, fused to the adjacent subarticular; outer metatarsal tubercle indistinct; foot webbing formula I2–2 1/2II1 1/2–2 3/4III1 1/2–2 1/3IV2 1/3–1 V; skin on dorsum, head, dorsal surfaces of forearms and thighs, flanks and groin smooth; skin on belly and ventral surfaces of thighs granular; cloacal opening directed posteriorly, covered by a dorsal sheath (vellum) from above; cloacal ornamentation absent.

### Measurements of holotype (in mm)

SVL: 16.5; head length: 5.1; head width: 6.0; eye diameter: 2.8; tympanum diameter: 1.0; eye-nostril distance: 1.4; hand length: 5.3; foot length: 6.6; thigh length: 7.5; shank length: 8.5.

### Holotype coloration in preservative

The general body coloration is pale yellow. Dorsal region dark brown colored, extending from the interorbital region towards the inguinal region. The dorsal colored area is irregular, with a narrowed area at shoulder girdle region, followed by an enlargement in the mid region of the body and an abrupt narrowing towards sacral area. The dorsolateral region is delimited by an irregular lighter brown (ocher) strip that extends from the posterior region of the eye to the inguinal area. The narrowing and enlargement of dorsal coloration is a reflex of the enlargement and narrowing of the dorsolateral strip, which is superiorly delimited by the dorsal colored area and inferiorly by a non-homogeneous dark brown line. The loreal region has the same color of dorsolateral strip and exhibit a triangular shaped mark delimited by a dark brown of dorsum in the interorbital region and by a darker brown line in the canthus rostralis. Arm, forearm, thigh and feet pale yellow. Dorsal surfaces of tibia with three dark brown bars. Ventral region homogeneously light cream colored.

### Variation in morphology and coloration

Measurements of the type series in Table 1. Finger disc of toe IV can be circular in some males. Alternative webbing formulae includes: I 2–2 II 11/2–21/2 III 2+–2+ IV and I 2–2+ II 11/2–21/3 III 11/2–21/3 IV 2+– 11/2 V. Females are larger than males (Fig 4). Morphology and color pattern are most of times concordant with the holotype, however, the degree of dorsal pigmentation varies greatly (Fig 4) from mostly dark brown to pale yellow with scattered dark spots (individual melanophores). The lighter dorsolateral strip almost always discernible (usually not homogeneously pigmented). The dorsolateral strip may be regular, without any enlargement or narrowing (as depicted in Fig 5B). Dorsum and limbs coloration varying from light brown to dark brown. The transversal bars on tibia posterior surface vary in size and thickness being more (Fig 5B) or less evident (Fig 5A and 5C); this feature may disappear in preserved specimens. Some specimens exhibit light colored spots below eyes (Fig 5A–5C). In living specimens, a light brown line outlines the eyes superiorly. Both females have better-defined pointed digit discs than males (Fig 3E and 3F).

**Table 1:** Measurement (in mm) for adult males and females of *Dendropsophus tapacurensis* sp. nov. type series.

Morphometric variables	Holotype (Male)	Male (*n* = 19)	Female (*n* = 2)
Snout-vent length	16.5	16.4 ± 0.6 (15.5–17.6)	20.1 ± 0.6 (19.5–20.7)
Head length	5.1	4.9 ± 0.5 (3.9–5.6)	5.8 ± 0.8 (5.1–6.6)
Head width	6.0	5.9 ± 0.3 (5.5–6.3)	6.8 ± 0.01 (6.8–6.8)
Eye diameter	2.8	2.5 ± 0.1 (2.4–2.8)	2.7 ± 0.06 (2.6–2.7)
Tympanum diameter	1.0	1 ± 0.03 (1.0–1.1)	1.2 ± 0.02 (1.2–1.2)
Eye-nostril distance	1.4	1.4 ± 0.02 (0.8–1.9)	1.6 ± 0.06 (1.5–1.6)
Hand length	5.3	5.4 ± 0.3 (4.8–5.8)	6.5 ± 0.4 (6.1–6.8)
Foot length	6.6	6.7 ± 0.6 (5.5–8.0)	8.8 ± 0.03 (8.5–9.0)
Thigh length	7.5	7.5 ± 0.5 (6.5–8.3)	9.5 ± 0.4 (8.9–9.6)
Shank length	8.5	8.5 ± 0.5 (7.0–8.9)	10.5 ± 0.3 (10.3–10.7)

Values are presented as mean ± SD (range). *n* = total number of measured individuals.

### Etymology

The specific epithet “*tapacurensis*” is to be treated as a noun in apposition and a direct reference to new species type locality, the Tapacurá Ecological Station. In *Tupi-guarani* (an indigenous South American linguistic family), the word "tapacura" (originally "*Itapacurá*") means: *I* = river; *ita* = rock; *pa* = spaciousness; *cura* = cover; meaning rock that covers the river or capped rock river. The suffix "ensis" is Latin and means "pertaining to" or "originating in".

### Comparisons with other species

The new species most resembles species of the *Dendropsophus decipiens* Group (as defined in [2]) in general morphology, call traits and color pattern. Morphometric comparisons in the *D*. *decipiens* Group are shown in Table 2.

**Table 2:** Measurement (in mm) for adult males of species in the *Dendropsophus decipiens* Group.

Variables	*D*. *berthalutzae* (*n* = 6)	*D*. *bromeliaceus* (*n* = 11)^*a*^	*D*. *decipiens* (*n* = 4)	*D*. *haddadi* (*n* = 8)	*D*. *oliveirai* (*n* = 6)	*D*. *tapacurensis* sp. nov. (*n* = 19)
Snout-vent length	20.4 ± 0.5 (20.0–21.2)	16.8 ± 6.6 (16.1–18.4)	17.4 ± 0.6 (16.7–18.4)	17.5 ± 0.6 (16.8–18.4)	14.3 ± 0.7 (13.6–15.5)	16.4 ± 0.6 (15.5–17.6)
Head length	7.1 ± 0.2 (6.8–7.5)	5.9 ± 3.5 (5.4–6.5)	5.9 ± 0.1 (5.8–6.0)	7.1 ± 0.4 (6.6–7.6)	5.5 ± 0.3 (5.2–6.0)	4.9 ± 0.5 (3.9–5.6)
Head width	6.5 ± 0.03 (6.0–7.0)	6.4 ± 2.3 (6.0–6.9)	5.2 ± 0.2 (5.0–5.5)	6.0 ± 0.2 (5.6–6.2)	4.0 ± 0.3 (4.7–5.5)	5.9 ± 0.3 (5.4–6.3)
Eye diameter	2.6 ± 0.02 (2.3–2.9)	1.8 ± 1.3 (1.7–2.1)	2.3 ± 0.2 (2.1–2.4)	2.5 ± 0.2 (2.2–2.7)	2.0 ± 0.1 (2.0–2.1)	2.5 ± 0.1 (2.4–2.8)
Tympanum diameter	1.6 ± 1.1 (0.8–1.8)	0.8 ± 1.4 (0.6–1.0)	0.8 ± 0.0 (0.7–0.8)	0.9 ± 0.2 (0.6–1.1)	0.9 ± 0.1 (0.7–1.0)	1 ± 0.03 (1.0–1.1)
Eye-nostril distance	1.8 ± 0.3 (1.4–2.1)	1.4 ± 1.4 (1.3–1.7)	1.7 ± 0.1 (1.6–1.9)	1.9 ± 0.1 (1.8–2.0)	1.5 ± 0.3 (1.1–1.9)	1.4 ± 0.02 (0.8–1.9)
Hand length	6.2 ± 0.02 (6.0–6.7)	–	5.3 ± 0.3 (5.0–5.7)	6.1 ± 0.5 (5.4–7.0)	4.8 ± 0.3 (4.3–5.0)	5.4 ± 0.3 (4.8–5.8)
Foot length	9.2 ± 0.3 (8.7–9.5)	7.0 ± 4.7 (6.1–7.5)	7.8 ± 0.3 (7.2–7.8)	8.5 ± 0.5 (7.8–9.3)	6.6 ± 0.4 (6.0–7.0)	6.7 ± 0.6 (5.5–8.0)
Thigh length	10.0 ± 0.4 (9.2–10.2)	8.3 ± 3.3 (7.8–8.6)	9.2 ± 0.4 (8.6–9.8)	9.8 ± 0.4 (9.0–10.5)	7.8 ± 0.2 (7.5–8.0)	7.5 ± 0.5 (6.5–8.3)
Shank length	10.3 ± 0.2 (10.1–10.5)	8.8 ± 4.6 (7.9–10.5)	9.6 ± 0.2 (9.5–10.0)	10.1 ± 0.4 (9.5–10.8)	7.2 ± 1.2 (5.5–8.6)	8.5 ± 0.5 (7.0–8.9)

See S1 Appendix for locality data of analyzed specimens.

^a^ Morphometric data of *D*. *bromeliaceus* retrieved from [4]. Hand length was not measured by authors (–).

*Dendropsophus tapacurensis* males (SVL = 15.5–17.6 mm) differs from those of *D*. *berthalutzae* (SVL = 20.0–21.2 mm) by being smaller, by the absence of a X-shaped mark on dorsum (present in *D*. *berthalutzae*), by the presence of the dorsolateral strip that extends from the posterior region of the eye to the inguinal region, and colored loreal region (absent in *D*. *berthalutzae*). From *D*. *decipiens* males (16.7–18.4 mm) by being slightly smaller (although values overlap), and by its slender body shape. From *D*. *haddadi* males (16.8–18.4 mm) by being slightly smaller (although values overlap) and by its slender body shape (also see acoustic comparisons and phylogenetic relationships). From *D*. *oliveirai* (white dorsolateral stripe), by the presence of light brown dorsolateral stripe. In general, specimens of *D*. *oliveirai* exhibit a darker brown coloration on dorsum; male specimens of *D*. *tapacurensis* are larger than those of *D*. *oliveirai* (13.6–15.5 mm). *Dendropsophus tapacurensis* males (ED = 2.4–2.8 mm) differs from those of *D*. *bromeliaceus* (ED = 1.7–2.1 mm) by a smaller eye, the absence of a cream mid-dorsal stripe from mid dorsum to cloaca, and by not using bromeliads for reproduction (*D*. *bromeliaceus* is the only bromeligenous species of the genus [4]).

Which respect to the calls (see advertisement call section), *Dendropsophus tapacurensis* differs from *D*. *haddadi* by its higher dominant frequency (5578–6422 Hz vs. 4312–4875 Hz in *D*. *haddadi* [16]), higher number of notes per series (3–9 notes in *D*. *tapacurensis* vs. 1–3 notes in *D*. *haddadi*), and longer note duration (39–110 ms in *D*. *tapacurensis* vs. 4–59 ms in *D*. *haddadi*). From *D*. *oliveirai* (56–161 pulses/s [46]) by its higher pulse rate (143–368 pulses/s) and pulses per note (5–14 in *D*. *oliveirai* vs. 9–29 in *D*. *tapacurensis*). From *D*. *decipiens* (4770–5230 Hz [47]) by its higher dominant frequency (5578–6422 Hz). From *D*. *berthalutzae* and *D*. *bromeliaceus* (complex call with two distinct notes; but see [48]) by emitting calls with one note type. In summary, the higher dominant frequency and notes with up to 29 pulses promptly distinguishes *D*. *tapacurensis* from its congeners of the *D*. *decipiens* Group (see Table 3 for a summary of advertisement calls parameters).

**Table 3:** Summary of acoustic parameters for species of the *Dendropsophus decipiens* Group.

Species	Call type	Notes per call	Pulses per notes	Note duration (ms)	Pulse rate	Dominant frequency (kHz)	Source
*D*. *berthalutzae* (*n* = 139)	simple call	1–9	2–6	10–96	#	3881–4294	[48]
*D*. *berthalutzae* (*n* = 32)	complex call	1–2	2–12	10–46	109–343	4315–4765	[49]
*D*. *bromeliaceus* (*n* = 28)	complex call	2	3–8	119–379	#	4800–5600	[4]
*D*. *decipiens* (*n* = 16)	simple call	4–11	#	20–130	#	4770–5230	[47]
*D*. *haddadi* (*n* = 86)	simple call	1–3	1–8	4–59	60–421	4312–4875	[16]
*D*. *oliveirai* (*n* = 28)	simple call	1	5–14	62–155	56–161	5685–6869	[46]
*D*. *tapacurensis* (*n* = 83)	simple call	3–9	9–29	39–110	143–368	5578–6422	**This work**

# = information not provided. *n* = total number of analyzed notes.

From other small species of *Dendropsophus*, formerly *D*. *microcephalus* Group (*sensu* [3]), *D*. *tapacurensis* differs from *D*. *anataliasiasi* (Bokermann, 1972), *D*. *araguaya* (Napoli and Caramaschi, 1998), *D*. *cachimbo* (Napoli and Caramaschi, 1999), *D*. *cerradensis* (Napoli and Caramaschi, 1998), *D*. *cruzi* (Pombal and Bastos, 1998), *D*. *elianeae* (Napoli and Caramaschi, 2000), *D*. *jimi* (Napoli and Caramaschi, 1999), *D*. *juliani* (Moravec et al. 2006), *D*. *rhea* (Napoli and Caramaschi, 1999), *D*. *rubicundulus* (Reinhardt and Lütken, 1862), *D*. *tritaeniatus* (Bokermann, 1965), *D*. *rozenmani* Jansen, Santana, Teixeira, and Köhler, 2019, *D*. *microcephalus* (Cope, 1886), *D*. *minusculus* (Rivero, 1971), *D*. *sanborni* (Schmidt, 1944), *D*. *walfordi* (Bokermann, 1962), *D*. *meridianus* (Lutz, 1954), *D*. *ozzyi* (Orrico et al., 2014), *D*. *shiwiarum* Ortega-Andrade and Ron 2013, *D*. *robertmertensi* (Taylor, 1937), *D*. *bipuncatus* (Spix, 1824), *D*. *studerae* (Carvalho-e-Silva et al., 2003), *D*. *branneri* (Cochran, 1948), *D*. *werneri* (Cochran, 1952), *D*. *reichlei* (Moravec et al., 2008), *D*. *gaucheri* (Lescure and Martin, 2000), *D*. *joannae* (Köhler and Lötters, 2001), *D*. *julianae* (Moravec et al., 2006), *D*. *coffeus* (Köhler et al., 2005), *D*. *mathiassoni* (Cochran and Goin, 1970), *D*. *sartori* (Smith, 1951), *D*. *phlebodes* (Stejneger, 1906), *D*. *rhodopelus* (Günther, 1858), *D*. *nanus* (Boulenger, 1889), *D*. *pseudomeridianus* (Cruz et al., 2000) and *D*. *riveroi* (Cochran and Goin, 1970) by the presence of dorsolateral stripes that extends from the posterior region of the eye to the inguinal region and colored loreal region (frame-like pattern, absent in all these species), except *D*. *gryllatus* (Duellman, 1973), currently not assigned to any species group [2], and *D*. *tintinnabulum* (Melin, 1941). However, *Dendropsophus tapacurensis* males differs from those of *D*. *gryllatus* (SVL = 22.6–25.5 mm) by its smaller SVL (15.5–17.6 mm in *D*. *tapacurensis*), and from *D*. *tintinnabulum* (note duration 10–21 ms with 2–4 pulses each [50]) by its longer note duration and greater pulse number per note (note lasting 39–110 ms with 9–29 pulses in *D*. *tapacurensis*).

### Advertisement call

**Fig 6:**
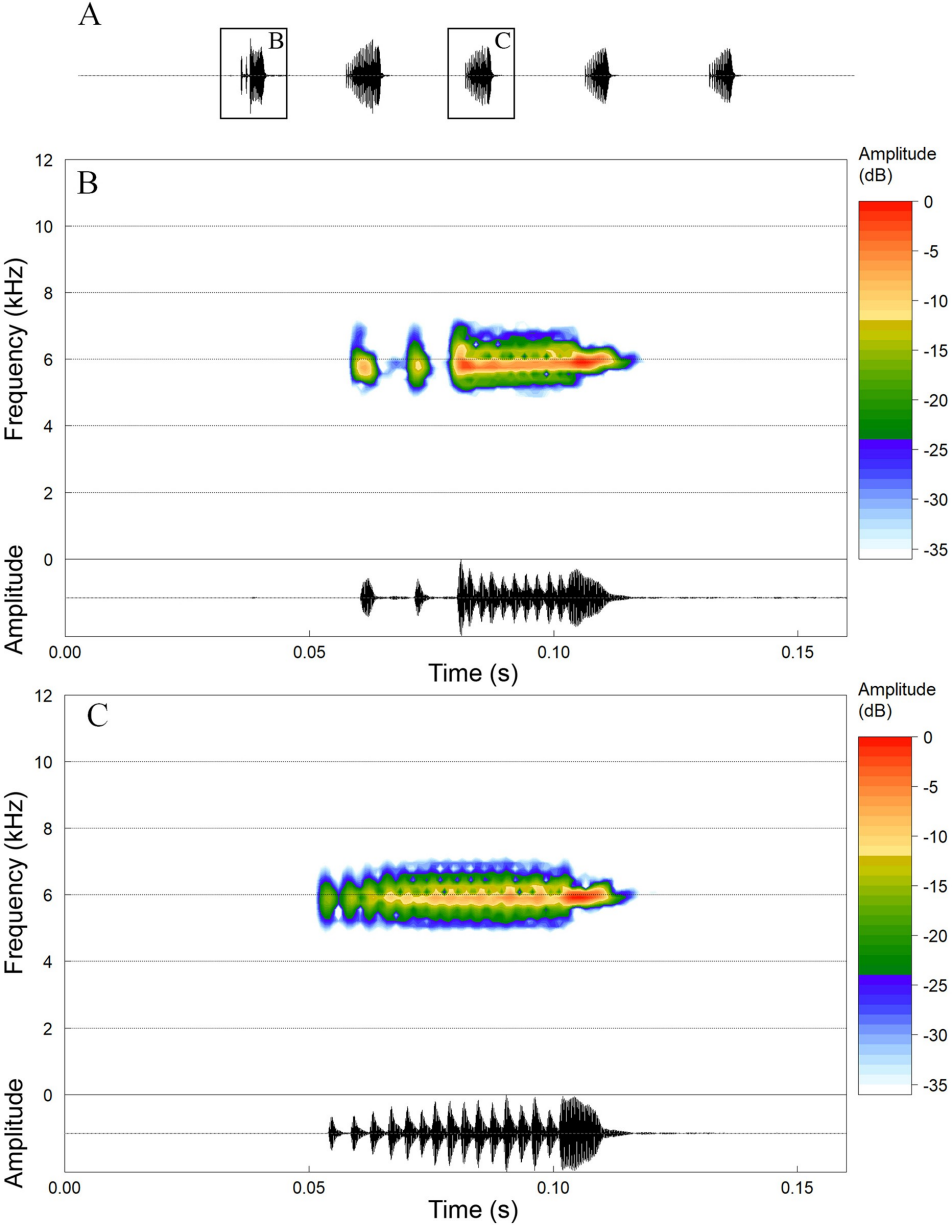
Advertisement call of *Dendropsophus tapacurensis* sp. nov. (A) waveform of a call series with five notes (two seconds section), the note outlined is detailed in the (B, spectrogram) and (C, waveform; sound file: SCLEHP22) Air temperature 24.5°C and humidity 72%.

The advertisement call of *D*. *tapacurensis* sp. nov. (Fig 6) is composed of one type of pulsed note emitted in series of 3–9 notes, or rarely isolated (*n* = 6 males; Table 3). The first note of a series may have between-pulse interval (Fig 6B), and the last pulse of each note is longer than the preceding ones. Notes lasting from 39–110 ms (mean: 62 ms; SD = 6.4; *n* = 83). Notes with 9–29 pulses (mean: 18.0; SD = 1.2; *n* = 83), pulse duration varies from 2 to 7 ms (mean 3.4 ms; SD = 0.3; *n* = 96), and pulse repetition rate from 143–368 pulses per second (mean 291; SD = 19.9; *n* = 83). Call group rate varies from 2.9 to 5.0 notes per second (mean 3.8; SD = 0.4; *n* = 29). Internote interval in the series varies from 146–364 ms (mean 201 ms; SD = 26.5; *n* = 67). Dominant frequency varies from 5578–6422 Hz (mean 5876 Hz; SD = 222.4; *n* = 83).

### Phylogenetics relationships

**Fig 7:**
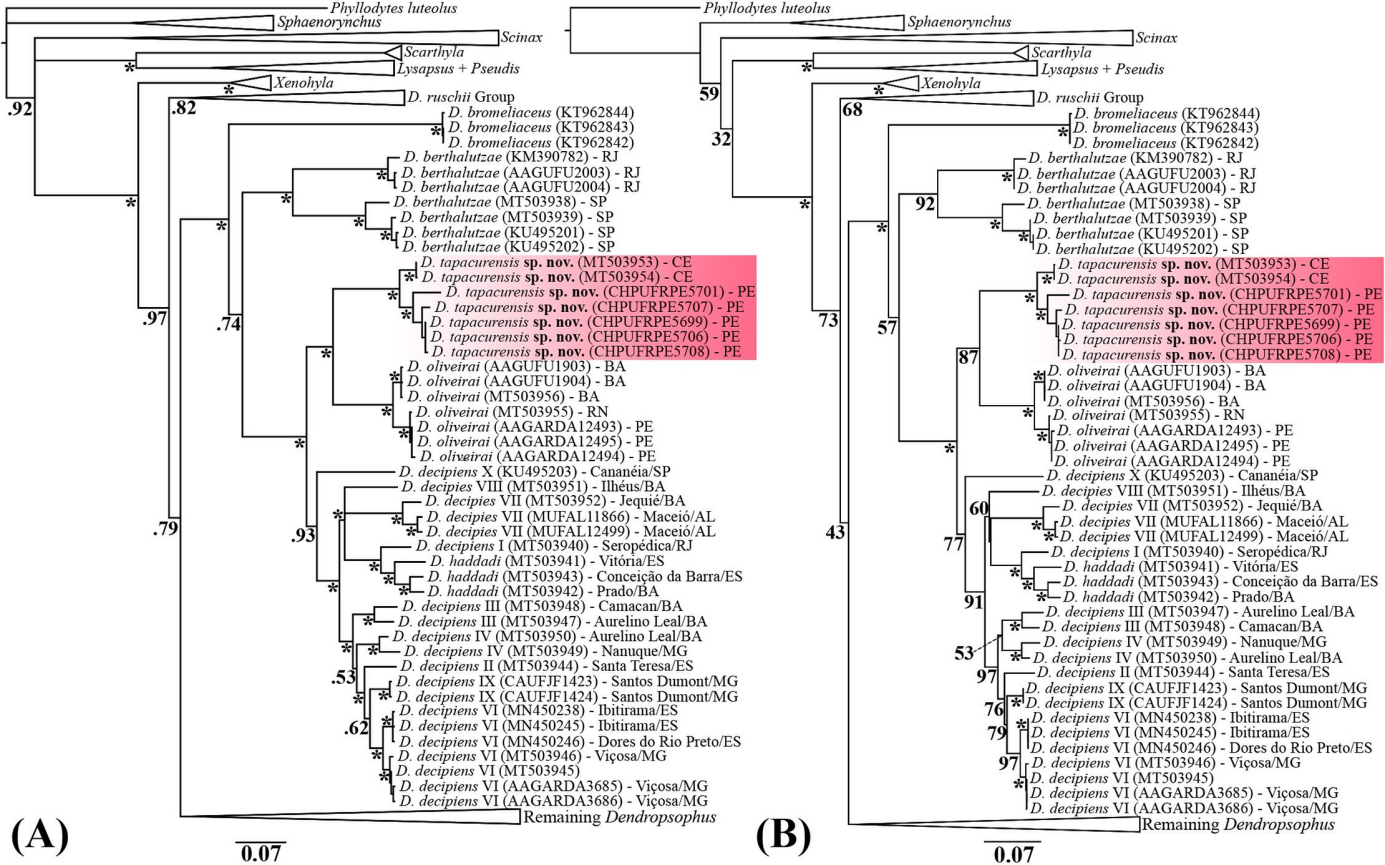
Phylogenetic relationship of *Dendropsophus tapacurensis* sp. nov. based on the mitochondrial *H1* dataset. (A) Bayesian and (B) maximum likelihood inferences. Numbers below nodes indicate posterior probability (Bayesian) and bootstrap scores (maximum likelihood). Asterisk indicate posterior probability/bootstrap scores = 1.0/100. Scale indicates rate of base substitutions per site.

Both Bayesian and maximum likelihood approaches yielded similar topologies, with major differences related to node support (Fig 7). The genus *Dendropsophus* was recovered as monophyletic and sister to *Xenohyla* with high/moderate node support (posterior probability [pp] = 0.97; bootstrap score [bs] = 73). The *D*. *decipiens* Group (*sensu* [2]) is strongly supported as monophyletic (pp = 1.0; bs = 100) with *D*. *bromeliaceus* appearing as the earlier divergent species within this Group. Subsequently, two deeply divergent lineages assigned to *D*. *berthalutzae* were recovered as the sister to all remaining species in the *D*. *decipiens* Group (pp = 0.74; bs = 57). The phylogenetic placement of *D*. *tapacurensis* sp. nov. within the *D*. *decipiens* Group as the sister taxon of *D*. *oliveirai* was recovered with high node support (pp = 1.0; bs = 87). Despite being morphologically more similar to *D*. *tapacurensis* sp. nov., terminals of *D*. *haddadi* (including topotypes) were recovered embedded among several genetically structured lineages of *D*. *decipiens* with high/moderate node support (pp = 0.93; bs = 77), remaining paraphyletic with respect to *D*. *haddadi*. In addition to eight *D*. *decipiens* clades uncovered by [2], we also highlight two other genetically structured *D*. *decipiens* clades from Santos Dumont, Minas Gerais State (CAUFJF1423–24) and from Cananéia, São Paulo State (GenBank sequence KU495203; voucher CFBHT07254), referred herein as lineages IX and X, respectively. It is noteworthy to mention that the paraphyly and lack of taxonomic resolutions of the *D*. *decipiens*-*D*. *haddadi* complex does not invalidate the hypothesis of our new species, considering its phylogenetic position within the *D*. *decipiens* Group as sister of *D*. *oliveirai*.

The average pairwise genetic distances for a 400 bp *16S* segment among species in the *D*. *decipiens* Group is very high (Table 4), especially if compared to the interspecific threshold of 3% proposed for Neotropical anurans [51]. For instance, if compared to all congeners in the *D*. *decipiens* Group, *D*. *tapacurensis* sp. nov. exhibit at least 8% of genetic distance (e.g., *D*. *oliveirai*; Table 4), while within-group distances do not exceed 3% (e.g., between *D*. *tapacurensis* populations from Ceará and Pernambuco States). Interestingly, lineages assigned to *D*. *decipiens* (I–X) exhibited higher levels of genetic diversity varying from 2% to up 11% for the *16S* gene. Such higher distances were also observed in sympatric lineages occurring at Aurelino Leal, Bahia State (e.g., III and IV; Fig 1), exhibiting 6% of genetic distance (Table 4). Accordingly, the newly uncovered lineage of *D*. *berthalutzae* from Rio de Janeiro State exhibited 9% of genetic distance in comparison with topotypical sequences from São Paulo State.

**Table 4:** Tamura-Nei corrected pairwise distances (average p-values) in the *Dendropsphus decipiens* Group estimated from the final 400 bp of the *16S* mitochondrial gene.

		1	2	3	4	5	6	7	8	9	10	11	12	13	14	15	within-groups
1	*D*. *tapacurensis* **sp. nov.**	–															0.03
2	*D*. *berthalutzae* (SP)	**0.14**	–														0.01
3	*D*. *berthalutzae* (RJ)	**0.13**	0.09	–													0.01
4	*D*. *bromeliaceus*	**0.14**	0.12	0.11	–												0
5	*D*. *decipiens* (I)	**0.10**	0.14	0.13	0.13	–											–
6	*D*. *decipiens* (II)	**0.11**	0.15	0.15	0.15	0.08	–										–
7	*D*. *decipiens* (III)	**0.10**	0.13	0.14	0.14	0.09	0.06	–									0.02
8	*D*. *decipiens* (IV)	**0.09**	0.14	0.13	0.14	0.06	0.05	0.06	–								0.03
9	*D*. *decipiens* (VI)	**0.09**	0.13	0.12	0.12	0.07	0.04	0.06	0.06	–							0.01
10	*D*. *decipiens* (VII)	**0.10**	0.14	0.14	0.15	0.07	0.07	0.08	0.08	0.07	–						0.02
11	*D*. *decipiens* (VIII)	**0.10**	0.12	0.13	0.13	0.06	0.07	0.08	0.07	0.05	0.08	–					–
12	*D*. *decipiens* (IX)	**0.09**	0.13	0.12	0.13	0.07	0.04	0.06	0.05	0.02	0.07	0.06	–				0
13	*D*. *decipiens* (X)	**0.10**	0.13	0.14	0.14	0.08	0.11	0.09	0.08	0.09	0.10	0.08	0.10	–			–
14	*D*. *haddadi*	**0.11**	0.14	0.14	0.14	0.05	0.07	0.08	0.07	0.06	0.08	0.06	0.06	0.08	–		0.02
15	*D*. *oliveirai*	**0.08**	0.13	0.14	0.12	0.09	0.11	0.10	0.09	0.09	0.10	0.09	0.08	0.08	0.08	–	0.01

Genetic distances between the new species and remaining species/lineages are highlighted in bold.

### Geographic distribution

*Dendropsophus tapacurensis* sp. nov. is only known from two areas along in Northeastern Brazil: (1) the type locality, municipality of São Lourenço da Mata, Pernambuco State, and (2) the municipality of Guaramiranga, Ceará State (Fig 1), a region characterized by relictual forest enclaves in high altitudinal areas (also known as *Brejo de altitude* or “wet islands”, reaching up to ~1100 m a.s.l. [52]) within the Baturité mountain range, located approximately 590 km northwest from the type locality.

### Natural history

**Fig 8:**
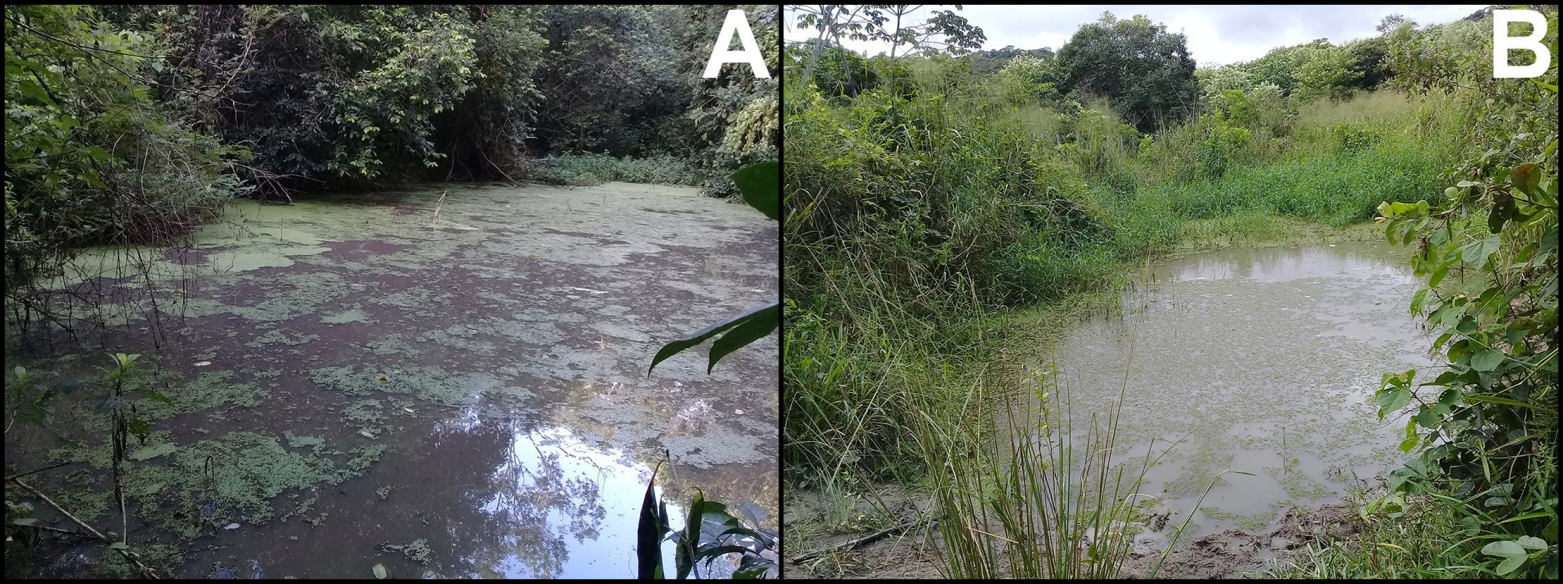
Temporary ponds where we found the new species in calling activity. (A) temporary pond within an Atlantic Forest strictly protected area; (B) temporary pond outside protected area amidst a sugar cane landscape.

We found specimens of *Dendropsophus tapacurensis* sp. nov. in calling activity on shrubs and marginal vegetation of temporary sandy bottomed ponds 15 to 100 cm deep (Fig 8). Males were commonly observed perched on leaves and branches at 10 to 150 cm from the ground. The species exhibits a prolonged breeding activity as calling males were heard during the entire rainy season (from April to August). Males usually start to call around 18:00 h and remain active until 23:00 h. The new species was found sympatrically with *Boana albomarginata*, *B*. *raniceps*, *Agalychnis granulosa*, *Scinax eurydice*, *S*. *auratus*, *S*. *pachycrus*, *Sphaenorhynchus prasinus*, *Leptodactylus natalensis*, *L*. *macrosternum*, *L*. *vastus*, *Physalaemus cuvieri* and *Dermatonotus mulleri*. Moreover, *D*. *tapacurensis* sp. nov. occurs syntopically with five other species of the genus *Dendropsophus*: *D*. *branneri*, *D*. *elegans*, *D*. *soaresi*, *D*. *minutus* and *D*. *oliveirai*.

### Remarks

Although *Dendropsophus tapacurensis* sp. nov. was only recorded at two sites along northeastern Brazil, there are several records attributed to *D*. *haddadi* in areas close to its type locality (Fig 1). Because *D*. *tapacurensis* sp. nov. and *D*. *haddadi* are considered morphologically cryptic species, it is plausible that records assigned to *D*. *haddadi* from Pernambuco and Alagoas might actually represent *D*. *tapacurensis* sp. nov., considering that the identification of these populations was based solely on external morphology. Therefore, the identity of these populations should be further investigated including acoustic and molecular evidence to better evaluate the distribution range and conservation status of *D*. *tapacurensis* sp. nov. Accordingly, considering that the Ceará population was attributed to *D*. *tapacurensis* based on DNA sequences, it is also important to confirm its taxonomic identity by relying on morphological and acoustic data in future contributions.

## Discussion

Within the *Dendropsophus decipiens* Group, *D*. *tapacurensis* sp. nov., most resembles *D*. *decipiens*, *D*. *haddadi* and *D*. *oliveirai*. The intraspecific variation of coloration patterns and body shape hampers discriminating these three species based solely on external morphology, specially based on a series of few individuals and/or long-time preserved ones. Apart from the *D*. *decipiens* Group, the presence of dorsolateral stripes that extends from the posterior region of the eye to the inguinal region and colored loreal region distinguishes *D*. *tapacurensis* sp. nov. from all species of *D*. *microcephalus* Group except *D*. *tintinnabulum* (Fig 1B of [50]), that may exhibit such pattern. The presence of discreetly pointed discs on finger (as in *D*. *tapacurensis* sp. nov.) was only reported for *D*. *shiwiarum* and *D*. *ozzyi* [53,54], which belongs to the *D*. *microcephalus* and *D*. *ruschii* Groups, respectively [2].

All species of the *D*. *decipiens* group have had its advertisement call described [4,16,46–49]. It is worth of note that *D*. *berthelutzae* has two separate calls descriptions. The description from [48] describes the species’ call based on males of five different populations (including topotypes) as being composed of one type of pulsed note, while [49] described the call from the municipality of Fervedouro, Minas Gerais State, as being composed of two pulsed notes named type “A” and “B”, suggesting a complex vocal repertoire. Because populations outside São Paulo State might correspond to undescribed taxa, a more detailed and standardized characterization of its calls are needed to understand call patterns within this Group. Three species of the *D*. *decipiens* Group have calls described from outside the type locality: *D*. *decipiens*, *D*.*oliveirai* and *D*. *haddadi* [16,46,47]. Considering the difficulty of identifying species of the *D*. *decipiens* Group based solely on external morphology and that some genetically structured lineages assigned to *D*. *decipiens* might correspond to new species, an acoustic review is of utmost importance to improve taxonomic resolutions within the *D*. *decipiens* Group.

In agreement to previous DNA-based and total-evidence phylogenetic analyses [2,3,55–58], we recovered the genus *Dendropsophus* as monophyletic with *Xenohyla* as its sister clade. Phylogenetic relationships within the *D*. *decipiens* Group overall agreed with that proposed by [2] based on phenomic and multilocus genetic datasets, except that *D*. *berthalutzae* was recovered as the earlier divergent species in this Group, and *D*. *bromeliaceus* the sister taxa to the remaining species (e.g., *D*. *decipiens*, *D*. *haddadi*, *D*. *oliveirai*). Such incongruences are likely related to differences in methodological approaches and datasets (e.g., we only used the mitochondrial *H1* segment). Nevertheless, the phylogenetic placement of *D*. *tapacurensis* sp. nov. as sister to *D*. *oliveirai* was congruent to that of ([2] labelled as “*D*. *decipiens* V”) and recovered with strong support in both Bayesian and likelihood inferences performed in our study. With the description of *D*. *tapacurensis* sp. nov., *D*. *decipiens* is no longer paraphyletic with respect to *D*. *oliveirai*, but remains paraphyletic to *D*. *haddadi*, as previously reported [2]. Moreover, we uncovered the existence of a deeply divergent genetic lineage assigned as *D*. *berthalutzae* and two additional lineages that clustered within the *D*. *decipiens*-*D*. *haddadi* complex, reinforcing that comprehensive morphological and acoustic data will be crucial to determine how many species exist in this *Dendropsophus* clade.

The new species displays a disjoint distribution along northeastern Brazil, with occurrence records at the type locality (coastal Atlantic Forest) and from Baturité mountain range (high-altitude relictual forest enclave). Both sites are typically composed by moist forests but with elevation difference ranging from 100 m (type locality) to approximately 1100 m (Baturité mountain range), being the last embedded within a semi-arid landscape. Nevertheless, the Baturité region harbors several relictual species/populations more related to Amazonian and Atlantic Forest species, including the frogs *Adelophryne baturitensis*, *Proceratophys renalis*, *Rhinella casconi*, and *R*. *gildae*, and *Scinax tropicalia* [59–61]. Such disjoint distribution and occurrence of forest-adapted species within this wet island environment is likely explained by historical connections between the Amazon and Atlantic rainforests that have crossed the interior of present-day Caatinga during Pleistocene (see [59,62]).

Most species of the *Dendropsophus decipiens* Group are strictly associated with the Atlantic Forest, which represents an important biogeographic region for studies focusing on the effects of Pleistocene climate changes on anuran genetic diversification [63–65]. More specifically, *D*. *haddadi* and *D*. *tapacurensis* sp. nov. are endemic to the Atlantic Forest, occurring along refuge areas that are well-known to harbor high levels of endemic species, such as the Central Corridor in Bahia State and the Pernambuco Endemism Center, from Alagoas to southern Paraiba States [63,66]. Although there are records for *D*. *haddadi* along Bahia and Pernambuco refuge zones (Fig 1), there are no studies that evaluated the genetic diversity and/or acoustic patterns of these populations, which were identified based solely on external morphology. Accordingly, we stress that some of these records might correspond to *D*. *tapacurensis* sp. nov., and showed that sequences identified as *D*. *haddadi* from Alagoas are genetically more related to the *D*. *decipiens* lineage VII of [2] than topotypical sequences of *D*. *haddadi*. We highly encourage future studies aiming to obtain additional data (especially covering along the Bahia Central Corridor) in order to elucidate the specific limits of other northeastern populations previously identified as *D*. *haddadi*, and to address whether the genetic structure and diversification of these populations/species agrees to the spatiotemporal expectations of Carnaval and Moritz model [63] for Atlantic Forest associated taxa, as previously reported for another *Dendropsophus* species [64].

Because of high rates of endemism and alarming levels of habitat loss in the Atlantic Forest (with approximately 11 to 16% of the original cover remaining [67]), this biodiversity hotspot is among the world`s top priorities for conservation [9]. Although the process of habitat loss occurred along the entire coastal region, it was more striking along northeastern Brazilian coast [10]. We emphasize the importance of Conservation Units, highlighting those along the Pernambuco Endemism Center, which shelters a high level of species diversity and endemism, and the potential for the discovery of additional unnamed species [68–70]. In addition, it should be noted as aggravating that this endemism center consists of small and highly fragmented Atlantic Forest remnants and therefore considered as the most threatened in Brazil [9,10,67].

## Supporting information

S1 Appendix Additional material examined for comparisons. (DOCX)

S2 Appendix Sound recordings and associated information. (PDF)

S3 Appendix GenBank accession number. Bold numbers are new sequences produced for this study.
(XLSX)
