# Supplementary material for: A new species of the Dendropsophus decipiens Group (Anura: Hylidae) from Northeastern Brazil
Source: PLoS One. 2021 Jul 14;16(7):e0248112. doi: 10.1371/journal.pone.0248112 (PMC8279364; doi:10.1371/journal.pone.0248112)
Supplement: S1 Appendix — (DOCX) [file pone.0248112.s001.docx]

**S1 Appendix. Additional material examined for comparisons.**

*Dendropsophus anataliasiasi*: type series: MZUSP 74204 (Ex. WCAB 45272); MZUSP-73790 (Ex. WCAB-45373); MZUSP 73788–73789 (Ex. WCAB 45257–45258); TOCANTINS: Brejinho de Nazaré (topotypes): AAG-UFU 0926–0939; Formoso do Araguaia: ZUEC 10158; ZUEC 10170–10171; ZUEC 10177–10178; ZUEC 101201; Nova Olinda: MNRJ 73004; MNRJ 66823.

*Dendropsophus araguaya*: type series: MZUSP 66803, MNRJ 17240–14241, MZUSP66796–66802, MZUSP 66719–66721, MNRJ 66796–66802; topotypes: AAG-UFU 1726–1724, MZUSP 152371–152380.

*Dendroposphus berthalutzae*: topotypes: AAG-UFU 5031–5038, AAG-UFU 0257–0259; SÃO PAULO: Ubatuba: AAG-UFU 4425–4426, Salesópolis: AAG-UFU 5410.

*Dendropsophus bipunctatus*: BAHIA: Ilhéus: AAG-UFU 0240; RIO DE JANEIRO: Macaé: AAG-UFU 0538–0540, Duas Barras: AAG-UFU 0702–0705, Cachoeiras de Macacu: AAG-UFU 6227; ESPÍRITO SANTO: Santa Teresa: AAG-UFU 6158–6160.

*Dendropsophus branneri*: BAHIA: Ilhéus: AAG-UFU 0241–0244, Prado: AAG-UFU 5971–5973; ESPÍRITO SANTO: Santa Teresa: AAG-UFU 6161–6163.

*Dendropsophus cachimbo*: type series: MNRJ 17298–17299; MZUSP 21910–21918; MZUSP 21920–21924; PARÁ: Novo Progresso: AAG-UFU 1489–1504; AAG-UFU 1506–1514 (topotypes); CHUNB 13098–13108; CHUNB 34445–34469; CHUNB 40178–40195.

*Dendropsophus cerradensis*: type series: MZUSP 06733, MNRJ 17293, ZUEC 06734–06737; MATO GROSSO DO SUL: Ribas do Rio Pardo: AAG-UFU 1709–1721.

*Dendropsophus cruzi*: type series: MNRJ 21791–21795; GOIÁS: Silvânia: AAG-UFU 1787–1792, AAG-UFU 5094–5097, Pirenópolis: AAG-UFU 0335–0339, Uruaçu: AAG-UFU 1009, AAG-UFU 1408, Alto Paraíso: AAG-UFU 1331, Minaçu: AAG-UFU 5053, Niquelândia: 5070; MATO GROSSO: Pontal do Araguaia: AAG-UFU 0218–0227, AAG-UFU 1087–1088, Alto Garças: AAG-UFU 5715–5717; MINAS GERAIS: Ituiutaba: AAG-UFU 0343–0348, AAG-UFU 0644–0645, Araporã: AAG-UFU 0657–0658, Limeira do Oeste: AAG-UFU 1755–1757; TOCANTINS: Brejinho de Nazaré: AAG-UFU 0917–0920, Mateiros: AAG-UFU 1987–1995, Palmas: AAG-UFU 2754–2758, AAG-UFU 3277–3292, MARANHÃO: Carolina: AAG-UFU 2840–2845.

*Dendropsophus decipiens*: RIO DE JANEIRO: Duas Barras: AAG-UFU 0706–0709.

*Dendropsophus elianeae*: MINAS GERAIS: Uberlândia: AAG-UFU 1891–1894, AAG-UFU 2294–2296, Sacramento: AAG-UFU 0895–0897, Perdizes: AAG-UFU 1030–1032; MATO GROSSO DO SUL: Bela vista (topotypes): AAG-UFU 0128–0142, Ribas do Rio Pardo: AAG-UFU 0160–0174, AAG-UFU 0655–0656.

*Dendropsophus haddadi*: ESPÍRITO SANTO: Santa Teresa: AAG-UFU 6170–6179, Sooretama: AAG-UFU 6203–6205.

*Dendroposphus jimi*: type series: MZUSP 21980, MNRJ 2198, MNRJ 21983–21990, MNRJ 21982, MINAS GERAIS: Araporã: AAG-UFU 0653–0654, Delta: AAG-UFU 0559–0562, Parque Nacional Grande Sertão Veredas: AAG-UFU 1907–1910, Uberlândia: AAG-UFU 2318–2327.

*Dendropsophus meridianus*: RIO DE JANEIRO: Manguinhos (topotypes): MNRJ 20761–20775; Cachoeiras de Macacu: AAG-UFU 6216–6225.

*Dendropsophus* aff*. microcephalus*: RONDÔNIA: Espigão d’Oeste: AAG-UFU 5798–5797.

*Dendropsophus nanus*: ARGENTINA: Resistencia (topotypes): UNNEC 12429–39; BRASIL: MINAS GERAIS: Araguari: AAG-UFU 0550–0552, Araporã: AAG-UFU 1825–1827, MATO GROSSO DO SUL: Bela vista: AAG-UFU 0159; TOCANTINS: AAG-UFU 0921–0925; MATO GROSSO: Cáceres: AAG-UFU 5255–5258; GOIÁS: Guarani de Goiás: AAG-UFU 1962; RONDÔNIA: Vilhena: AAG-UFU 5268, AAG-UFU 5362–5364.

*Dendropsophus oliveirai*: type series: MZUSP 7359, MZUSP 74146-74148; BAHIA: Maracás: 5673-5679.

*Dendropsophus ozzyi*: type series: MZUSP 154084–154088.

*Dendropsophus pseudomeridianus*: RIO DE JANEIRO: Macaé: AAG-UFU 0548–0549, AAG-UFU 758–760, MINAS GERAIS: AAG-UFU 1103.

*Dendropsophus rhea*: type series: MZUSP 9104, MZUSP 30983–30984, MZUSP 14450–14471, MZUSP 9105–9113; SÃO PAULO: Pirassununga: topotypes: AAG-UFU 1089–1097.

*Dendropsophus rodhopeplus*: EQUADOR: Santa Cecília: MZUSP 55812–55821, BRASIL: Rondônia: Itapuã do Oeste: AAG-UFU 5816–5819.

*Dendropsophus rubicundulus*: MINAS GERAIS: Lagoa Santa (topotypes): AAG-UFU 0021–0022, Parque Nacional da Serra do Cipó: AAG-UFU 0030–0044, Vargem Bonita: AAG-UFU 0605, São Gotardo: AAG-UFU 1749–1754, Buritis: AAG-UFU 1754–1768, Curvelo: AAG-UFU 0306, Juatuba AAG-UFU 0327, Parque Nacional Grande Sertão Veredas: AAG-UFU 1895-1906; Paracatu: AAG-UFU 0647–0650; MATO GROSSO: Cuiabá: 1447–1461; MARANHÃO: Carolina AAG-UFU 2833–2839; GOIÁS: Guarani de Goiás: AAG-UFU 1953–1961; Silvânia AAG-UFU 1785–1786, Uruaçu: AAG-UFU 1003–1008, Teresina de Goiás: AAG-UFU 1355–1379, Padre Bernardo: AAG-UFU 1526–1535; TOCANTINS: Mateiros: AAG-UFU 2149, 2160–2162; Paranã: AAG-UFU 3293–3305; BAHIA: São Desiderio AAG-UFU 5434–5436.

*Dendropsophus sanborni*: URUGUAI: MALDONADO: Maldonado: CH-UR 17632–17636, ZUEC 10073–10077; SAN JOSÉ: Ciudad del Plata: MZUSP 77997–98, MZUSP 78000, MZUSP 78002, MZUSP 78006–78009, MZUSP 78001–78012; CERRO LARGO: Plácido Rosas: MZUSP 7761–7764; BRASIL: SÃO PAULO: Botucatu: AAG-UFU 1704–1708, Águas da Prata 162 AAG-UFU 3526–3527, Itatiba: AAG-UFU 4431; MINAS GERAIS: Poços de Caldas: AAG-UFU 4680–4681, AAG-UFU 0001–0002.

*Dendropsophus tritaeniatus*: MATO GROSSO: Chapada dos Guimarães: AAG-UFU 1467–1474.

*Dendropsophus walfordi*: type series: MZUSP 73652 (ex WCAB 8436), MZUSP 74019–74041, MZUSP 74424, MZUSP 73653; RONDÔNIA: Costa Marques (topotypes) AAG-UFU 5306–5327, MZUSP 129903–130122, Itapuã do Oeste: AAG-UFU 5820–5829; Abunã: MZUSP 104496–104503; ACRE: Rio Branco: AAG-UFU 5886–5894, Feijó: AAG-UFU 5894, Cruzeiro do Sul: AAG-UFU 5903–5906, AMAZONAS: Borba: MZUSP 51199–51212.

*Dendropsophus tintinnabulum*: AMAZONAS: São Gabriel da Cachoeira: AAG-UFU 3854–3857, 3882–3884; Tarauacá: MZUSP: 188212–188213.
