# Supplementary material for: A new species of the Dendropsophus decipiens Group (Anura: Hylidae) from Northeastern Brazil
Source: PLoS One. 2021 Jul 14;16(7):e0248112. doi: 10.1371/journal.pone.0248112 (PMC8279364; doi:10.1371/journal.pone.0248112)
Supplement: S2 Appendix — (PDF) [file pone.0248112.s002.pdf]

S2 Appendix. Sound recordings and associated information.

Sound file	Species	Voucher	Locality (State)	Air temperature/humidity
SCLEHP18	*D. tapacurensis* sp. nov.	Unvouchered	São Lourenço da Mata (PE)	23.8ºC/73%
SCLEHP19	*D. tapacurensis* sp. nov.	CHP_UFRPE5697	São Lourenço da Mata (PE)	23.8ºC/73%
SCLEHP20	*D. tapacurensis* sp. nov.	Unvouchered	São Lourenço da Mata (PE)	23.9ºC /74%
SCLEHP21	*D. tapacurensis* sp. nov.	CHP_UFRPE5704	São Lourenço da Mata (PE)	23.9Cº /74%
SCLEHP22	*D. tapacurensis* sp. nov.	CHP_UFRPE5705	São Lourenço da Mata (PE)	24.5Cº/72%
SCLEHP23	*D. tapacurensis* sp. nov.	CHP_UFRPE5706	São Lourenço da Mata (PE)	24.8ºC/78%
SCLEHP24	*D. tapacurensis* sp. nov.	CHP_UFRPE5707	São Lourenço da Mata (PE)	22.9ºC/85%
SCLEHP25	*D. tapacurensis* sp. nov.	CHP_UFRPE5708	São Lourenço da Mata (PE)	23.8ºC/73%
SCLEHP26	*D. tapacurensis* sp. nov.	CHP_UFRPE5709	São Lourenço da Mata (PE)	22.9ºC/79%
SCLEHP27	*D. tapacurensis* sp. nov.	CHP_UFRPE5710	São Lourenço da Mata (PE)	23.5ºC/76%
SCLEHP28	*D. tapacurensis* sp. nov.	CHP_UFRPE5711	São Lourenço da Mata (PE)	23.9ºC/74%
